# Highly Sensitive Immunosensing of Carcinoembryonic Antigen Based on Gold Nanoparticles Dotted PB@PANI Core-Shell Nanocubes as a Signal Probe

**DOI:** 10.1155/2023/7009624

**Published:** 2023-04-07

**Authors:** Dexiang Feng, Lingzhi Chen, Ke Zhang, Shuangshuang Zhu, Meichen Ying, Peng Jiang, Menglan Fu, Yan Wei, Lihua Li

**Affiliations:** ^1^Department of Chemistry, Wannan Medical College, Wuhu 241002, China; ^2^Institute of Synthesis and Application of Medical Materials, Department of Pharmacy, Wannan Medical College, Wuhu 241002, China

## Abstract

Herein, a method was developed for the sensitive monitoring of carcinoembryonic antigen (CEA) by gold nanoparticles dotted prussian blue@polyaniline core-shell nanocubes (Au NPs/PB@PANI). First, a facile low-temperature method was used to prepare the uniform PB@PANI core-shell nanocubes with the assistance of PVP, where PB acted as the electron transfer mediator to provide electrochemical signals, and the PANI with excellent conductivity and desirable chemical stability not only played the role of a protective layer to prevent etching of PB in basic media but also effectively improved electron transfer. Importantly, to further enhance the electrical conductivity and biocompatibility of PB@PANI and to further enhance the electrochemical signal and capture a large amount of Ab_2_, Au NPs were doped on the surface of PB@PANI to form Au NPs/PB@PANI nanocomposites. Subsequently, benefiting from the advantages of core-shell structure nanoprobes and gold-platinum bimetallic nanoflower (AuPt NF), a sandwich-type electrochemical immunosensor for CEA detection was constructed, which provided a wide linear detection range from 1.0 pg·mL^−1^ to 100.0 ng·mL^−1^ and a low detection limit of 0.35 pg·mL^−1^ via DPV (at 3*σ*). Moreover, it displayed a satisfactory result when the core-shell structure nanoprobe-based immunosensor was applied to determine CEA in real human serum samples.

## 1. Introduction

As we all know, carcinoembryonic antigen (CEA) is a tumor marker for colon cancer, breast cancer, ovarian cancer, and other cancers, which can provide reliable information for the early diagnosis and treatment of tumor patients [1–3]. Consequently, the highly sensitive determination of CEA is a pressing need by virtue of accurate and efficient analytical techniques. Immunosensors based on antibody-antigen interaction are one of the most widely used analytical techniques in the quantitative detection of biomarkers [4]. Among them, electrochemical immunosensors have attracted much attention due to their characteristics of high specificity, good sensitivity, short time consumption, low energy consumption and trace detection, and more suitable for the detection of biomarkers in low concentration [5, 6]. More recently, lots of electrochemical immunosensors have been constructed for quantitation of CEA. For example, Wang et al. Jozghorbani et al. and Wang et al. designed three kinds of label-free CEA immunosensors with detection limits of 0.005, 0.05, and 0.0429 ng·mL^−1^, respectively [7–9]. Especially, a large number of reports have focused on the sandwich-type electrochemical immunosensor for CEA detection [10–13], thanks to their distinct advantages such as lower background noise, higher sensitivity, and importance, and they have higher selectivity for analytes through two specific reactions, which are superior to label-free counterparts [14–16].

Prussian blue (PB) with a face-centered cubic lattice structure is popular as a kind of electrochemical redox-active species in electrochemical biosensors because of its high electrochemical/electrocatalytic properties and low redox potential [17, 18]. Unfortunately, the poor stability and low conductivity of PB limit its further applications in biosensors [19]. To minimize these problems, some conducting materials can be introduced to meet the requirements mentioned above. Polyaniline (PANI) as one of the most desired materials with good chemical stability, low cost, and good electrical conductivity can not only provide a conductive substrate but also can form an effective coating layer on “the core” such as carbon nanotubes (CNTs) or PB [20, 21]. The combination of PB and PANI (PB@PANI) can play a significant synergistic effect and improve the electrocatalytic performance, conductivity, and stability [21]. Furthermore, to further enhance the conductivity and biocompatibility of PB@PANI, gold nanoparticles (Au NPs) were introduced over the surface of PB@PANI by Au-N bonds between Au NPs and -NH_2_ from PANI to form Au NPs/PB@PANI nanocomposites [15]. To the best of our knowledge, the preparation and application of Au NPs/PB@PANI nanocomposites in electrochemical immunosensors have not been reported. Hence, the PB@PANI loaded with Au NPs (Au NPs/PB@PANI) would be a promising nanoprobe.

For sandwich-type electrochemical immunosensors, to achieve signal amplification and high sensitivity, it is a more important key to effectively immobilize the primary antibody (Ab_1_) [22, 23]. Gold and platinum nanoparticles are the most promising bimetallic materials for various applications due to their superior biocompatibility, higher specific surface area, and superior electrocatalytic properties towards the reduction of H_2_O_2_ [24–26]. They can be decorated onto the surface of the L-cysteine (L-Cys)-modified electrode to provide an available microenvironment for loading amounts of Ab_1_, thus greatly improving the sensitivity of the immunosensor.

This work was developed by electrodeposition of AuPt bimetallic nanoflower (AuPt NFs) on the GCE modified with L-Cys as a sensing platform and Au NPs/PB@PANI as a novel label prepared by a facile low-temperature method. The existence of AuPt NFs not only fixed the primary antibody but also accelerated the electron transfer. Furthermore, the as-developed Au NPs/PB@PANI nanoprobes were used as a tracer for the generation and amplification of electrochemical signals and easily captured second antibodies (Ab_2_) via Au-N bond. As expected, a sandwich-type electrochemical immunosensor capable of achieving large signal amplification was developed by connecting Ab_1_-AuPt NFs/L-Cys with Ab_2_-Au NPs/PB@PANI.

## 2. Materials and Methods

### 2.1. Materials and Chemicals

Carcinoembryonic antigen (CEA) and its antibodies, *α*-fetoprotein (AFP), were purchased from Biocell Biotech. Co., Ltd. (Zhengzhou, China). Chloroauric acid (HAuCl_4_·4H_2_O), chloroplatinic acid (H_2_PtCl_6_ 6H_2_O), bovine serum albumin (BSA), polyvinyl pyrrolidone (PVP), L-cysteine (L-Cys), aniline, ammonium persulfate, and so on were purchased from Aladdin Reagent Co., Ltd. (Shanghai, China).

### 2.2. Devices

Electrochemical measurements were made at the CHI 660E electrochemical workstation (Shanghai Co., Ltd., China). The morphology of nanomaterials was obtained by a scanning electron microscopy (SEM, JSM-7100F, Japan) and a transmission electron microscopy (TEM, JEM-6700F, Japan).

### 2.3. Synthesis of PB Nanocubes

PB nanocubes were synthesized according to a previous work despite a minor modification [27]. 8.0 mmoL·L^−1^ Na_4_Fe(CN)_6_.8H_2_O, 4.0 mL hydrochloric acid (37%), and 2.0 g polyvinyl pyrrolidone (*M*_*w*_∼40000) were added into deionized water (400 mL) and stirred for 30 min, and then were refluxed at 60°C for 6 h. After that, the blue products were washed with deionized water and dried in vacuum at 100°C for 24 h.

### 2.4. Preparation of PB@PANI Core-Shell Nanocubes

PB@PANI core-shell nanocubes were synthesized with reference to previous work with slight variations [21]. Briefly, 0.6 g·PB, 0.5 g polyvinyl pyrrolidone (*M*_*w*_∼40000) and 100 *μ*L aniline monomer were dissolved in 100 mL hydrochloric acid (1 moL·L^−1^) with sonication for 1 h. Then, ammonium persulfate solution (1.6 mmoL·L^−1^) was added dropwise into the above solution and stirred for 18 h in an ice bath. Finally, the PANI-coated PB (core-shell structure PB@PANI) was collected by centrifugation and washed with deionized water and ethanol for several times.

### 2.5. Preparation of Ab_2_-Au NPs/PB@PANI Bioconjugates

15 mg PB@PANI nanocubes were added into the concentrated Au NPs colloidal solution (5.0 mL, 1.0 mg·mL^−1^) and reacted 30 min under shaking. Au NPs were assembled on the surface of PB@PANI to form the Au NPs/PB@PANI nanocomposites by the chemical bond of Au-NH_2_ [15]. Followed by centrifugation and washing twice with deionized water, the obtained precipitate was redispersed into PBS solution (pH 7.0). Subsequently, 500 *μ*L CEA-Ab_2_ (2.0 mg·mL^−1^) was added into the Au NPs/PB@PANI suspension (2 mL) and stirred for 12 h at 4°C. To get rid of nonspecific adsorption, 100 *μ*L of 1% BSA was mixed with the obtained conjugates for 6 h at 4°C. Finally, the bioconjugates were centrifuged and redispersed in PBS (pH 7.0, 10 mmoL·L^−1^), and then stored at 4°C.

### 2.6. Fabrication of the Immunosensor

**Scheme 1:**
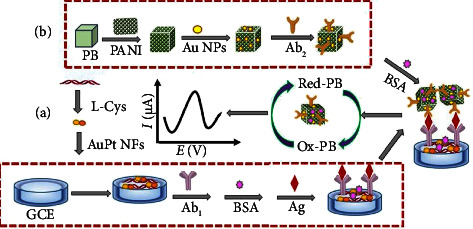
(a) The construction process of electrochemical immunosensor. (b) Assembly diagram of immunoprobes.

For polymer nanocomposite film deposition, polished glass carbon electrode (GCE) was first immersed in 0.5 mmoL·L^−1^ cysteine containing PBS (pH 7.0) and was scanned in the potential range of −0.0 to 1.7 V at 50 mV/s for 3 cycles. The obtained electrode was dried in air, then dipped into 0.2 moL·L^−1^ H_2_SO_4_ and the solution containing 0.5 mmoL·L^−1^ HAuCl_4_ and 0.5 mmoL·L^−1^ H_2_PtCl_6_, and electrodeposited with chronoamperometric method at −0.2 V vs. Ag/AgCl for 600 s at room temperature. After rinsing, the AuPt NFs/L-Cys/GCE-modified electrode was immersed in primary antibody (CEA-Ab_1_) solution at 4°C overnight, and Ab_1_ was connected to the modified electrode. Next, to remove excess binding sites, the modified electrode was immersed in 1 wt% BSA solution at room temperature for 40 min. Finally, the obtained electrode was washed three times with PBS and stored at 4°C.  Scheme 1(a) displays the schematic of the designed immunosensor, and Scheme 1(b) shows the preparation procedure of Ab_2_-Au NPs/PB@PANI bioconjugates.

### 2.7. Electrochemical Detection

The prepared immunosensors were incubated in the solutions of CEA antigen with different concentrations for 40 min and then continued incubated in Ab_2_-Au NPs/PB@PANI solution at 37°C for the same time. Subsequently, differential pulse voltammetry (DPV) and electrochemical impedance spectroscopy (EIS) were performed in PBS (pH 6.5) and 5 mmoL·L^−1^ [Fe (CN)_6_]^3-/4-^, respectively.

## 3. Results and Discussion

### 3.1. Characterization of the Nanomaterials

**Figure 1:**
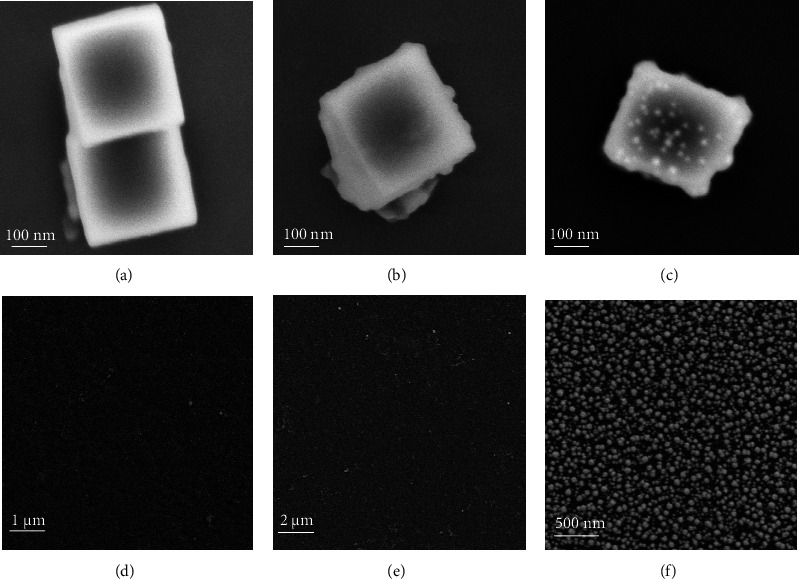
(a) SEM images of PB, (b) PB@PANI, (c) Au NPs-PB@PANI, (d) GCE, (e) L-Cys/GCE, and (f) AuPt NFc/L-Cys/GCE.

SEM images of the PB, the PB@PANI, and the Au NPs/PB@PANI are displayed in Figures 1(a)–1(c). The as-prepared PB nanocomposites were made up of cubes with side lengths in the range of 300–320 nm (Figure 1(a)), which had a rough surface to provide abundant nucleation sites for the uniform growth of PANI shell [28]. It was clearly seen that the nanocubes of PB were completely covered by the PANI shell, and the thickness of the PANI was about 10 nm after the reaction time of 18 h (Figure 1(b)). When Au NPs with diameters of about 15 nm were attached to the surface of PB@PANI, we clearly observed that Au NPs with uniform globular morphology were absorbed on the surface of PB@PANI core-shell nanocubes (Figure 1(c)), which can also immobilize CEA-Ab_2_ through Au-N bonds.

Figures 1(d) and 1(e) show the morphology of the electrode. As exhibited in Figure 1(d), the surface of the bare GCE was not smooth. By electrodeposition, the L-Cys film was deposited on the surface of GCE (Figure 1(e)), which maybe influence on the size and crystalline structure of AuPt bimetallic nanoflowers (AuPt NFs) according to previous reports [29]. At the same time, the presence of L-cysteine helped to bind the AuPt NFs on the surface of GCE. Quasi-spherical flower-like AuPt NFs with diameters of about 50 nm were observed after the electrodeposition on L-Cys/GCE (Figure 1(f)).

Meanwhile, Au, Pt, C, N, and S elements were found in the energy dispersive spectroscopy (EDS) of AuPt NFs/L-Cys/GCE (Figure S1A). EDS can further prove that Au NPs/PB@PANI nanocomposites were composed of Au, Pt, K, N, C, O, and Fe elements (as shown in Figure S1B).

### 3.2. Characterization of the Constructed Immunosensor

**Figure 2:**
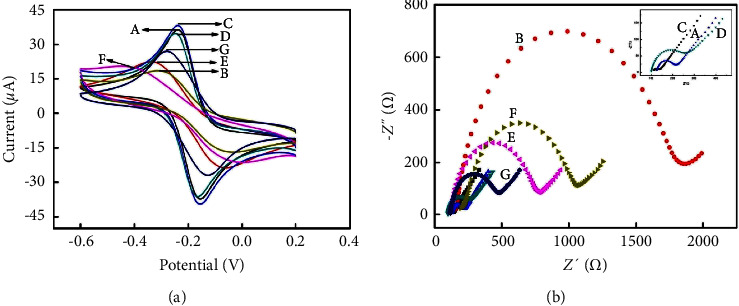
(a) CVs and (b) EIS of (A) bare GCE, (B) L-Cys/GCE, (C) AuPt NFs/L-Cys/GCE, (D) Ab1/AuPt NFs/L-Cys/GCE, (E) BSA/Ab1/AuPt NFs/L-Cys/GCE, (F) Ag/BSA/Ab1/AuPt NFs/L-Cys/GCE, (G) Bioconjugates/Ag/BSA/Ab1/AuPt NFs/L-Cys/GCE in [Fe(CN)_6_]^3-/4-^.

CV and EIS measurements were utilized to monitor the stepwise assembly process of the immunosensors [30]. As shown in Figure 2(a), the peak current of L-Cys film modified GCE electrode by one-step electropolymerization was decreased clearly (curve b) compared with the bare GCE electrode (curve a), indicating L-Cys film hindered electron transfer. In addition, modification of AuPt NFs onto the film of L-Cys caused an increase in peak current benefiting from excellent conductivity of the AuPt NFs (curve c). After Ab_1_ being adsorbed on the modified electrode (curve d), blocking with BSA (curve e), and modifying with Ag (curve f), a further decrease in the peak currents was seen, due to the effect of biomacromolecules hindering electron transport. However, the peak currents increased significantly after the modified electrodes were combined with Ab_2_-Au NPs/PB@PANI bioconjugates (curve g), indicating that Ab_2_-Au NPs/PB@PANI bioconjugates can greatly enhance electron transfer. Moreover, EIS results of each step of electrode modification were in accordance with those of CV.

### 3.3. Optimization of Experimental Conditions

**Figure 3:**
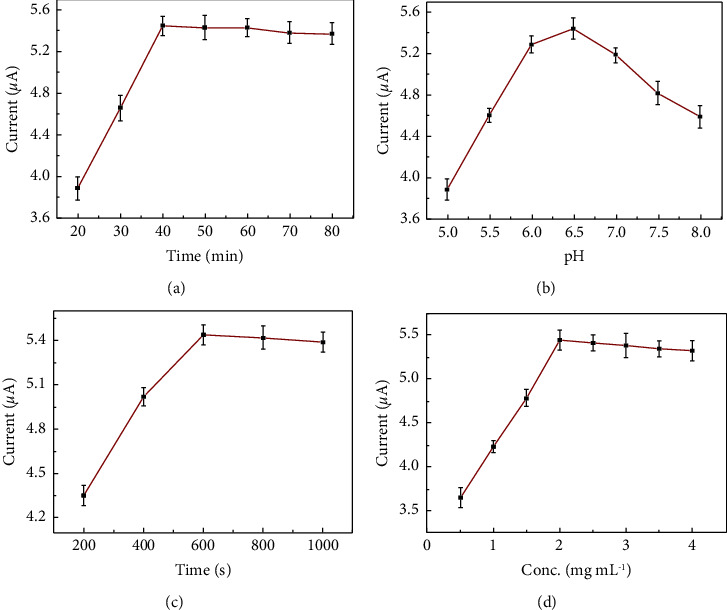
Influence of (a) incubation time, (b) pH, (c) deposition time of AuPt NFs, and (d) the concentration Au NPs/PB@PANI on the peak currents to *C*_CEA_ = 10.0 ng·mL^−1^. Error bars represent standard deviations from five repeated measurements.

To improve the sensitivity and analysis efficiency of the proposed immunosensor, the experimental conditions were optimized. As shown in Figure 3(a), when incubation time was 20 min–80 min, the DPV response increased gradually and then reached a plateau after 40 min because the combination of the antigen with Ab_1_ reached equilibrium in approximately 40 min.

The pH value of buffer solution has a significant influence on the stability and activity of electrochemical biosensors. As can be seen from Figure 3(b), with the change of pH, the signal of DPV response also changed. In the range of pH 5.0–8.0, DPV value first increased and then slightly decreased. Au NPs/PB@PANI nanoprobes possessed optimal activity and stability in approximately neutral or acid environment due to an effective coating layer of conducting PANI. Therefore, we chose PBS solution with a pH of 6.5 as the appropriate electrolyte.

In the meantime, the deposition time of AuPt NFs had a significant effect on DPV response as well. As displayed in Figure 3(c), as the deposition time of AuPt NFs increased from 200 to 1000 s, the current signal increased gradually and then reached a plateau at 600 s. Therefore, the deposition time of AuPt NFs was selected as 600 s for the preparation of the immunosensor.

Similar to reported biosensor, the performance of immunosensor was highly affected by the concentration of Au NPs/PB@PANI. As shown in Figure 3(d), with the increasing concentration from 0.5 mg·mL^−1^ to 2.0 mg·mL^−1^, the current response increased rapidly, but when the concentration was higher than 2.0 mg·mL^−1^, the current response reached a plateau. Therefore, 2.0 mg·mL^−1^ became the optimum concentration for this study.

### 3.4. Quantitative Detection of CEA

**Figure 4:**
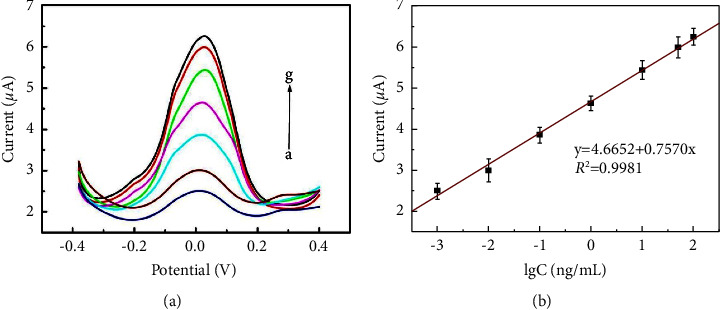
(a) DPV responses of the as-prepared immunosensor after incubation with CEA concentrations (From a–g: 0.001, 0.01, 0.1, 1.0, 10.0, 50.0, and 100 ng·mL^−1^) in 0.1 M PBS (pH 6.5). (b) Calibration curves of the immunosensor. Error bars represent standard deviations from five repeated measurements.

Under optimal experimental conditions, CEA was quantified by using the developed immunosensor. As shown in Figure 4, the DPV signal increased gradually as the CEA concentration increased. There was a good linear relationship between the current intensity and the logarithm of CEA concentration in the range of 0.001–100 ng·mL^−1^. The regression equation was *I*_*P*_(*μ*A) = 4.6652 + 0.7570*x* (*R*^2^ = 0.9981) with 0.35 pg·mL^−1^ detection limit. In comparison with other nanoprobe-based immunosensors [31–37], the detection limit of the prepared immunosensor was lower (Table 1). The reasons for the excellent analytical performance of the designed immunosensor may be summarized as follows: first, PANI, as the conductive shell of PB@PANI core-shell structure, maintained the structural stability of PB and effectively improved the electron transfer from PANI to PB due to the intimate adhesion. Second, a large amount of Au NPs distributed on the PB@PANI surface provided a large specific surface area for the binding of antibodies, which enhanced the immobilization of CEA-Ab_2_. In addition, the Au NPs/PB@PANI nanoprobes with the unique structure and synergetic contributions exhibited good conductivity and high stability, increased the conduction efficiency of signal molecules, and can be used as a signal amplification system to improve the sensitivity of detection. Finally, AuPt NFs with multifunctionality were used as an ideal sensing platform, not only can capture more Ab_1_ and improve conductivity but also constituted a dual signal amplification system together with Au NPs/PB@PANI nanoprobes.

### 3.5. Specificity, Stability, and Reproducibility of the Immunosensor

**Figure 5:**
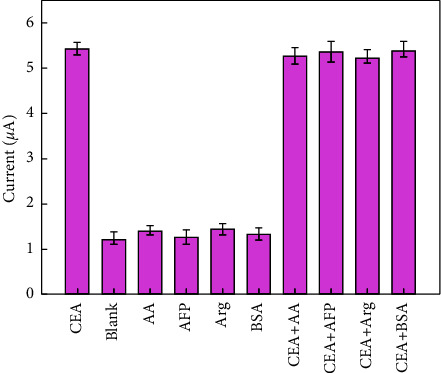
The specificity of the CEA immunosensor. Error bars = SD (*n* = 5).

In order to investigate the specificity of the constructed immunosensor targeting CEA, 100.0 ng·mL^−1^ of four interferences including alpha fetoprotein (AFP), ascorbic acid (AA), glucose (Glu), and BSA was added to CEA (1.0 ng·mL^−1^) solution, respectively. As displayed in Figure 5, these interferences made a neglectful influence to the target, suggesting the proposed biosensor presented high specificity for the specific recognition of CEA.

**Figure 6:**
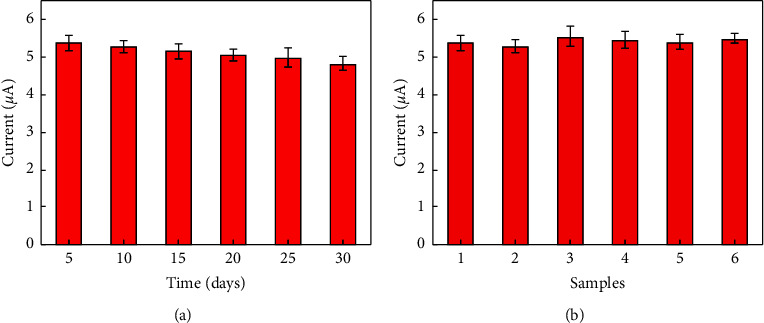
(a) The stability and (b) reproducibility of the CEA immunosensor. Error bars = SD (*n* = 5).

The stability experiments of the immunosensor were carried out by storing several immunosensors at 4°C for 30 days, which were tested for CEA every 5 days (Figure 6(a)). After 30 days, the DPV value retained 88.6% of its initial response. The effective fixation of Ab_2_ on the Au NPs/PB@PANI nanoprobe and the efficient protection of PB in the PANI coating made the developed immunosensor have high stability.

To further verify the reproducibility of the immunosensor, six immunosensors were incubated in 10.0 ng·mL^−1^ of CEA for DPV determination. As shown in Figure 6(b), the RSD was 3.1% after measurement, demonstrating the reproducibility of the immunosensor was acceptable.

### 3.6. Determination of Real Serum Sample

To certify analytical reliability and application potential of the developed immunosensor, ten clinical serum specimens containing CEA were analyzed. As displayed in Table 2, the relative error between the two methods was in the range from −5.26% to 6.94%, and these data revealed that the developed immunosensor had good practicability for clinical serum analysis.

## 4. Conclusions

In conclusion, an electrochemical immunosensor based on gold nanoparticles functionalized PB@PANI core-shell nanocubes as a signal amplification strategy was fabricated for CEA detection. The high sensitivity of the immunosensor was attributed to the reasons as follows. First, the abundant N-species in PANI can be coupled with PB to effectively protect the structural degradation of PB in basic media. Second, Au NPs/PB@PANI nanocubes containing abundant electron mediators provided a large signal of DPV. Finally, AuPt NFs were dotted on the L-Cys/GCE electrode to increase the specific surface area and electrical conductivity, which not only immobilize more antibodies but also accelerate the electron transfer. Moreover, the designed immunosensor showed the especial merit that avoided the addition of mediators to the buffer or separated the immobilization of mediators onto the electrode. Promisingly, the strategy can be easily extended to the application scope of Au NPs/PB@PANI nanocomposites.

## Figures and Tables

**Table 1:** Comparison of analytical performance of CEA immunosensors with different nanoprobes.

Nanoprobes	Linear range (ng·mL^−1^)	Detection limit (ng·mL^−1^)	Reference
Ag-BSA-Pt NPs	0.005–100.00	7.60 × 10^−4^	[31]
CPS@PANI@Au	0.006–12.00	1.56 × 10^−3^	[32]
PTh-Au	0.3–30.00	1.47 × 10^−4^	[33]
3D-rGO-MWCNTs/Ag-Au Ps	0.001–80.00	3.00 × 10^−3^	[34]
AuNPs-PAN@CNTs	0.002–80.00	8.00 × 10^−3^	[35]
CNSs@Au NPs	0.002–80.00	3.00 × 10^−3^	[36]
Ce-MoF@HA/Ag-HRP	0.001–80.00	2.00 × 10^−4^	[37]
AuNPs-PB@PANI	0.001–100.00	3.50 × 10^−4^	This work

**Table 2:** Comparison with ELISA for CEA.

Serum sample no.	This method (ng·mL^−1^)	ELISA^a^ (ng·mL^−1^)	RSD (%)
1	0.06 ± 0.01	0.058 ± 0.02	+3.45
2	0.54 ± 0.12	0.57 ± 0.08	−5.26
3	1.88 ± 0.14	1.85 ± 0.05	+1.62
4	5.00 ± 0.22	5.14 ± 0.13	−2.72
5	13.26 ± 0.42	13.76 ± 0.57	−3.63
6	18.78 ± 1.23	17.56 ± 1.65	+6.94
7	23.55 ± 1.09	24.06 ± 0.79	−2.12
8	37.67 ± 0.78	36.87 ± 1.52	+2.17
9	50.33 ± 1.77	52.26 ± 0.67	−3.69
10	78.38 ± 1.39	75.97 ± 1.83	+3.17

^a^Mean value ± SD of five measurements.

## Data Availability

The data supporting this study are from previously reported studies and datasets, which have been cited.

## Supplementary Materials

Supplementary Materials For the characterization of the signal probe material, we added the energy dispersive spectroscopy (EDS) as supplementary material, provided with the manuscript. Figure S1. EDX spectra of (A) AuPt NFs/L-Cys/GCE and (B) Au NPs/PB@PANI.
