# Supplementary material for: Highly Sensitive Immunosensing of Carcinoembryonic Antigen Based on Gold Nanoparticles Dotted PB@PANI Core-Shell Nanocubes as a Signal Probe
Source: J Anal Methods Chem. 2023 Apr 7;2023:7009624. doi: 10.1155/2023/7009624 (PMC10104734; doi:10.1155/2023/7009624)
Supplement: Supplementary Materials — For the characterization of the signal probe material, we added the energy dispersive spectroscopy (EDS) as supplementary material, provided with the manuscript. Figure S1. EDX spectra of (A) AuPt NFs/L-Cys/GCE and (B) Au NPs/PB@PANI. [file 7009624.f1.pdf]

Electronic Supplementary Material

Highly sensitive immunosensing of carcinoembryonic antigen based on gold nanoparticles dotted PB@PANI core-shell nanocubes as a signal probe

Dexiang Feng,[1,2] Lingzhi Chen,[2] Ke Zhang,[1,2] Shuangshuang Zhu,[1] Meichen Ying,[2] Peng Jiang,[2] Menglan Fu,[2] Yan Wei,[1,2] Lihua Li[2]

[1]Department of Chemistry, Wannan Medical College, Wuhu, 241002, China

[2]Institute of Synthesis and Application of Medical Materials, Department of Pharmacy, Wannan Medical College, Wuhu, 241002, China

Correspondence should be addressed to Lihua Li; llh05530226@126.com and Yan Wei; yanwei@wnmc.edu.cn

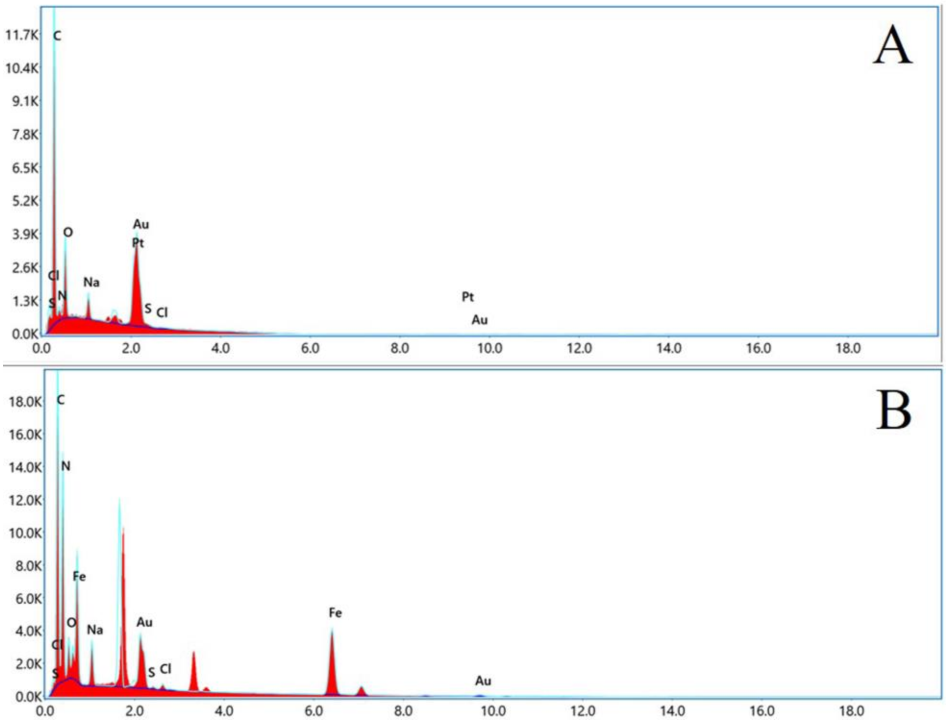

FIGURE. S1 EDX spectra of (A) AuPt NFs/L-Cys/GCE, (B) Au NPs-PB@PANI.
